# Supplementary material for: The causal relationship between the human gut microbiota and pyogenic arthritis: a Mendelian randomization study
Source: Front Cell Infect Microbiol. 2024 Nov 26;14:1452480. doi: 10.3389/fcimb.2024.1452480 (PMC11629706; doi:10.3389/fcimb.2024.1452480)
Supplement: Supplementary file 1 [file DataSheet1.zip › mendelian test/ebi-a-GCST90027666.csv_leave_one_out.pdf]

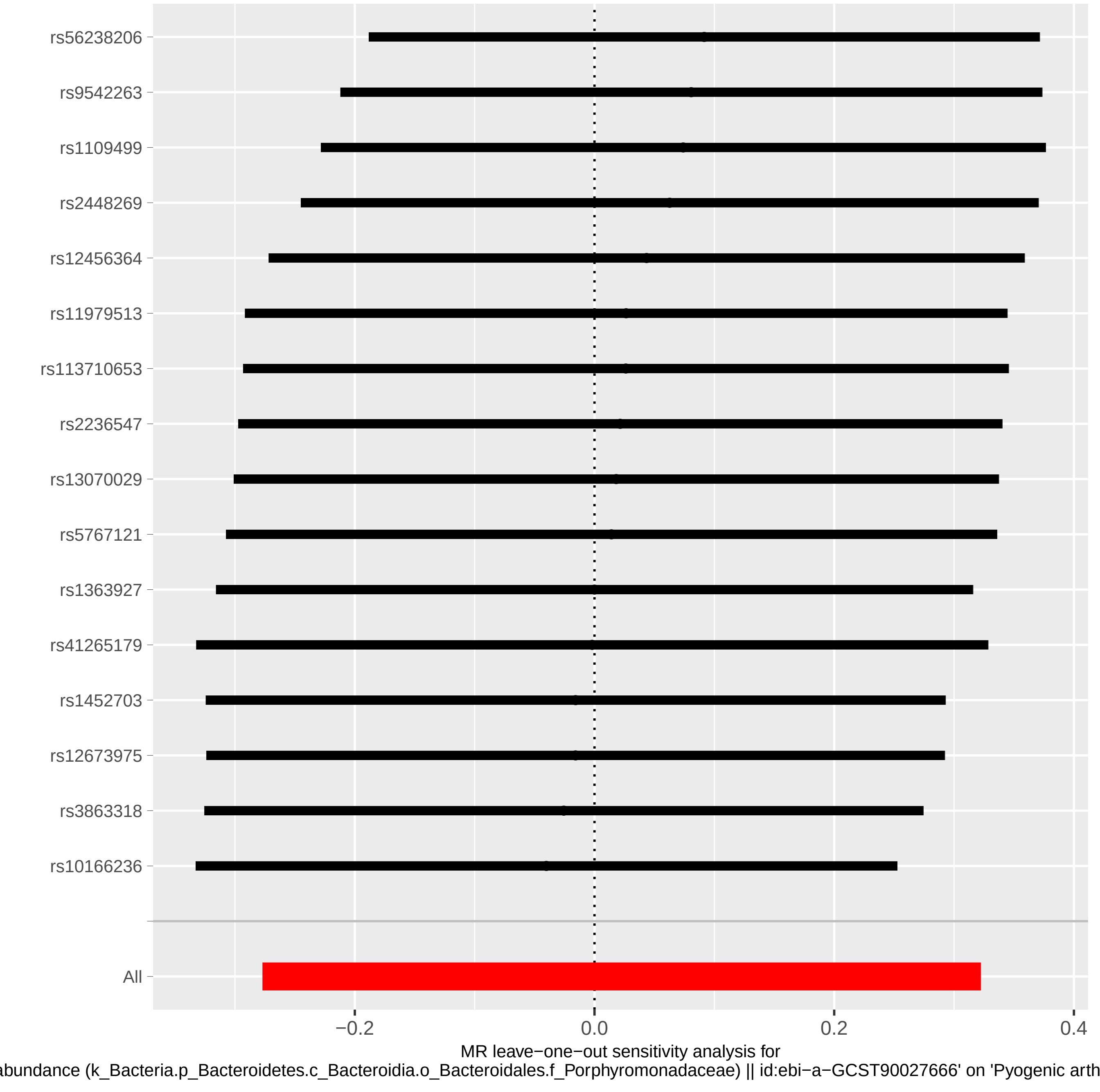
rs56238206
rs9542263
rs1109499
rs2448269
rs12456364
rs11979513
rs113710653
rs2236547
rs13070029
rs5767121
rs1363927
rs41265179
rs1452703
rs12673975
rs3863318
rs10166236
All
−0.2
0.0
0.2
0.4
MR leave−one−out sensitivity analysis for
abundance (k_Bacteria.p_Bacteroidetes.c_Bacteroidia.o_Bacteroidales.f_Porphyromonadaceae) || id:ebi−a−GCST90027666' on 'Pyogenic arth
